# Downregulation of *STOX1* is a novel prognostic biomarker for glioma patients

**DOI:** 10.1515/biol-2021-0119

**Published:** 2021-10-25

**Authors:** Fei-qin Jin, Lei Jin, Yan-ling Wang

**Affiliations:** Department of Radiology, People’s Hospital of Gaoxin District, Suzhou, 215129, Jiangsu, China; Department of Neurosurgery, Guangdong Provincial People’s Hospital, Guangdong Academy of Medical Sciences, Guangzhou, 510000, Guangdong, China; Department of Surgery, Division of Neurosurgery, Li Ka Shing Faculty of Medicine, the University of Hong Kong, Hong Kong, China

**Keywords:** bioinformatics, glioma, *STOX1*, overall survival, biomarker

## Abstract

*Storkhead box 1 (STOX1)* is a winged helix transcription factor structurally and functionally related to the forkhead family of transcription factors. Recent studies have highlighted its role in the central nervous system and revealed hints in the development of glioma. However, the expression profiles of *STOX1*, its association with clinicopathological characteristics, and potential functions in glioma remain unknown. In this study, we analyzed three publicly available datasets including CGGA, TCGA, and Rembrandt and revealed a grade-dependent reduction in *STOX1* expression in glioma (*P* < 0.001). Chi-square test demonstrated that low *STOX1* expression was significantly associated with older age at initial diagnosis (*P* < 0.001), less IDH1 mutation (*P* < 0.001), and advanced WHO grade (*P* < 0.001). Moreover, multivariate Cox regression analysis showed that *STOX1* expression may serve as a novel independent prognostic biomarker in glioma patients. Bioinformatic functional analysis (GSEA) predicted that *STOX1* was related to many key cancer pathways including P53 signaling pathway (*P* < 0.01), DNA replication (*P* < 0.05), homologous recombination (*P* < 0.05), and Wnt signaling pathway (*P* < 0.05). Taken together, these findings suggested that *STOX1* may be used as a novel predictive molecular biomarker for glioma grading and overall patient survival. Further investigations on the functional roles and therapeutic value of *STOX1* in glioma are warranted.

## 1 Introduction

Glioma accounts for the majority (around 46%) of malignant tumors in the central nervous system, with glioblastoma multiforme (GBM) bearing the highest degree of malignancy [1,2]. Although significant advances have been made over the past few decades in treatment, including surgery, radiotherapies, chemotherapies, and biotherapies, the median survival of glioma patients remains largely unchanged, with that of GBM patients being limited to 12–14 months [1,2,3]. It has been well established that many biological and molecular factors are involved in the pathogenesis, progression, and therapy response of glioma [4], and the identification of novel biomarkers continues to generate potential therapeutic targets.

*Storkhead box 1* (*STOX1*), also known as an open reading frame on human chromosome 10 (C10PRF24), is located on 10q22.1 and encodes a winged helix transcription factor closely related to the Forkhead protein family [5]. *STOX1* was originally described to consist of six isoforms: A, B, C, D, E, and F, by alternative splicing [6,7]. Two of them, *STOX1A* and *STOX1B*, have been more thoroughly studied. *STOX1A* is the most complete isoform of *STOX1* encompassing a DNA-binding domain and a transactivator domain, while *STOX1B* shares only the former [5,7], suggesting a possible competition between these two isoforms in regulating DNA expression. Most functional studies on *STOX1* have so far focused on *STOX1A* in regards to its involvement in multiple biological processes, including cell cycle [8,9], early development [10,11], and oxidative stress regulation [12]. *STOX1A* also plays important roles in multiple diseases. Extensive studies have reported its substantial role in preeclampsia disorders such as gestational hypertension and proteinuria [6,7]. Abundant expression of *STOX1* has been observed in the brain, suggesting that *STOX1* may contribute to brain homeostasis and that *STOX1* dysregulation may lead to diseases. Indeed, *STOX1A* is upregulated and closely related to the late onset and the severity of Alzheimer’s disease [13], and *STOX1* may also function as a transcriptional suppressor of Math1 during cerebellar granule neurogenesis and medulloblastoma formation [14]. Retroviral gene-trap and functional characterization of primary mouse astrocyte clones under p53^–/–^ background found that *STOX1A* was one of the top two altered proteins, suggesting loss of *STOX1* might contribute to gliomagenesis [15]. However, the role of *STOX1* in glioma remain largely unknown.

In this study, we, for the first time, explore the expression profile and prognostic value of *STOX1* in glioma patients using public datasets including the Chinese Glioma Genome Atlas (CGGA), The Cancer Genome Atlas (TCGA), and Repository of Molecular Brain Neoplasia Data (Rembrandt). Due to the lack of transcriptome datasets that distinguish between transcript isoforms, the expression profiles of *STOX1* as a single entity were assessed. The signaling pathways that may involve *STOX1* and which are relevant to glioma pathogenesis were analyzed through GSEA. We found that low *STOX1* expression would predict a higher degree of glioma malignancy and worse overall patient survival, and that *STOX1* may serve as a potential molecular target for glioma.

## 2 Materials and methods

### 2.1 Data source and presentation

The clinical information and the *STOX1* mRNA expression microarray data were obtained from CGGA (325 adult gliomas, http://cgga.org.cn), TCGA (609 adult gliomas, https://www.cancer.gov), and Rembrandt (344 adult gliomas, http://caintegrator.nci.nih.gov/rembrandt). The glioma datasets from the Affymetrix U133.0 plus 2.0 platform were normalized using the Robust Multi-Array Average algorithm implanted in the R statistical software. Edge R Packets implanted in the R statistical software were used to normalize the TCGA-GBM and TCGA-LGG (low-grade glioma) data from RNA sequencing. The patients were divided into low- and high-expression groups according to the median value of *STOX1* expression in each dataset for further analysis.

### 2.2 Statistical analysis

All data analyses were performed by IBM SPSS 23.0 software (SPSS Inc. Chicago, IL, USA) and GraphPad Prism 7.0 (GraphPad Software, San Diego, CA). Differences in the *STOX1* mRNA expression levels between two groups or among multiple groups were evaluated by student’s *t*-test or one-way analysis of variance (ANOVA) followed by Dunnett *post hoc* test, respectively. Overall survival analysis was performed using the Kaplan–Meier method and log-rank (Mantel–Cox) test. The Pearson’s chi-square test was employed to assess the distribution of patient’s characteristics between subgroups. Univariate and multivariate Cox proportional hazard regression analyses were carried out to evaluate the prognostic value of *STOX1* expression, among other factors. The gene sets regulated by *STOX1* were predicted by the Kyoto Encyclopedia of Gene Set Enrichment Analysis (GSEA). Tests were 2-tailed, and *P* < 0.05 was considered statistically significant.

## 3 Results

### 3.1 Correlations between *STOX1* expression and the glioma grade

**Figure 1:**
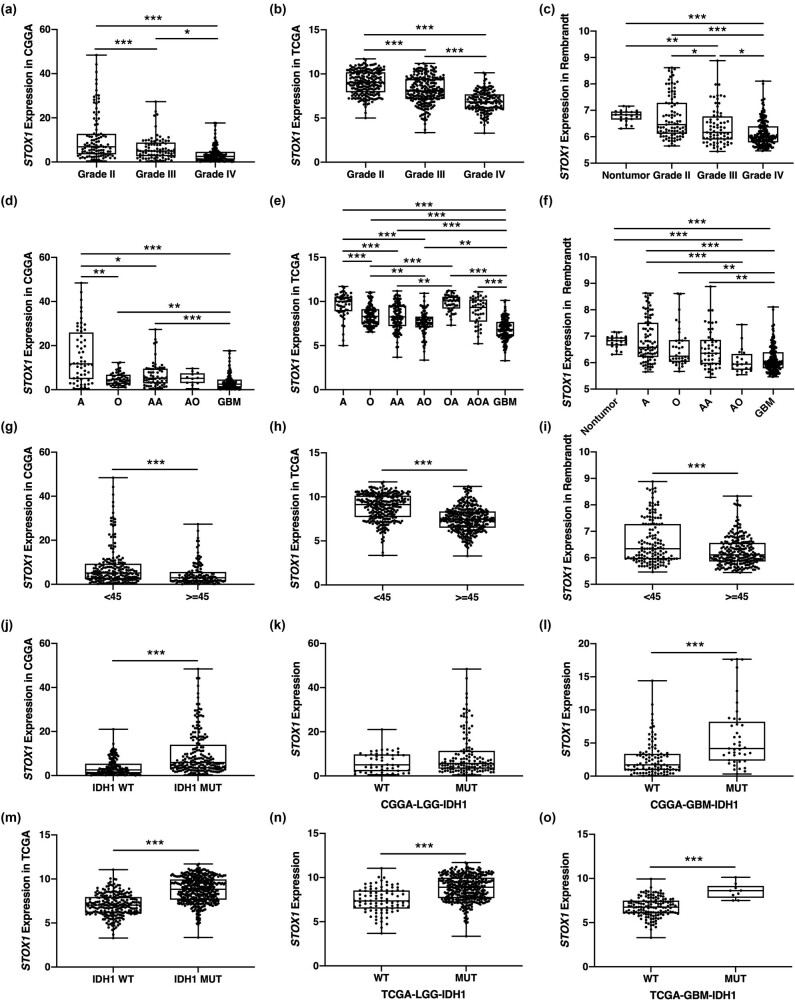
*STOX1* expression was correlated with glioma grade. (a and c) *STOX1* expression in different WHO grades of gliomas in CGGA (a), TCGA (b), and the Rembrandt (c) datasets. (d–f) *STOX1* expression in different histological types of gliomas in CGGA (d), TCGA (e), and Rembrandt (f) datasets. (g and i) *STOX1* expression in different age groups (<45 years and ≥45 years) in CGGA (g), TCGA (h), and Rembrandt (i) datasets. (j–l) *STOX1* expression in IDH1-WT and -MUT gliomas (j), LGGs (k), and GBMs (l) in CGGA dataset. (m–o) *STOX1* expression in IDH1-WT and -MUT gliomas (m), LGGs (n), and GBMs (o) in TCGA dataset. *P < 0.05; **P < 0.01; ***P < 0.001. A: astrocytomas; O: oligodendroglioma; AA: anaplastic astrocytoma; AO: anaplastic oligodendroglioma; OA: oligoastrocytoma; AOA; anaplastic oligoastrocytoma; GBM: glioblastoma multiforme; LGG, low-grade glioma; IDH1, isocitrate dehydeogenase 1; WT: wild type; MUT: mutant; CGGA: Chinese Glioma Genome Atlas; TCGA: The Cancer Genome Atlas; Rembrandt: repository of molecular brain neoplasia data; WHO, World Health Organization.

To determine the expression profiles of *STOX1* in different grades of gliomas, the CGGA-325 dataset (Grade II, *n* = 103; Grade III, *n* = 79; and Grade IV, *n* = 139) was first analyzed. As shown in Figure 1a, *STOX1* expression showed a grade-dependent reduction (II vs III, *P* < 0.001; II vs IV, *P* < 0.001; and III vs IV, *P* = 0.019) as tumor grade increases. To verify this finding, the TCGA dataset (Grade II, *n* = 216; Grade III, *n* = 241; and Grade IV, *n* = 152) was also analyzed and demonstrated that *STOX1* expression was evidently associated with WHO grading (II vs III, II vs IV, and III vs IV, all *P* < 0.001) (Figure 1b). To further validate these results, the *STOX1* mRNA expression profiles in the Rembrandt dataset were then analyzed. As expected, *STOX1* expression showed a grade-dependent trend, although no significant difference was observed between grade II gliomas and their non-tumor counterparts (Figure 1c). To further determine the *STOX1* expression pattern in glioma, we analyzed *STOX1* expression in different histological types of gliomas in each dataset. Consistent with the above results, *STOX1* expression in GBMs was consistently lower than that in astrocytomas, oligodendrogliomas (Grade II and III), and nontumor brain tissues (Figure 1d–f). To determine *STOX1* expression in different age groups, glioma patients in each dataset were separated into <45- and ≥45-years groups. We found that *STOX1* expression was significantly lower in older patients in all three datasets (Figure 1g–i).

**Figure A1:**
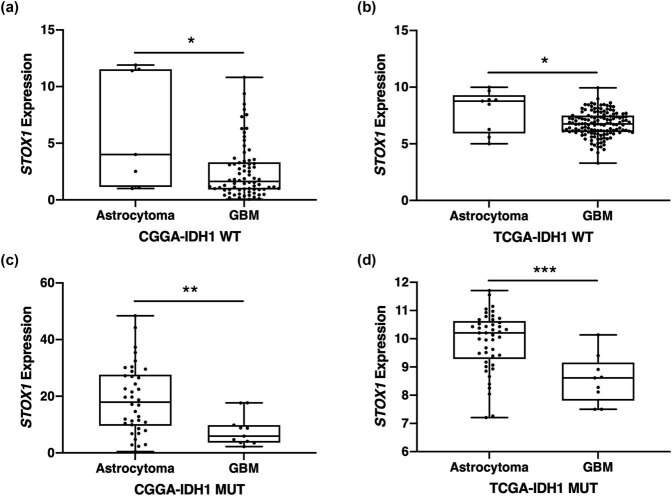
*STOX1* expression in GBM was higher than that in astrocytoma. (a and b) *STOX1* expression in IDH1-WT astrocytomas and GBMs of the CGGA and TCGA datasets. (c and d) *STOX1* expression in IDH1-MUT astrocytomas and GBMs of the CGGA and TCGA datasets. *P < 0.05; **P < 0.01; ***P < 0.001. GBM: glioblastoma multiforme; WT: wild type; MUT: mutant; CGGA: Chinese Glioma Cancer Atlas; TCGA: The Cancer Genome Atlas.

Molecular features such as mutations in IDH1, ATRX, and P53 and codeletion of chromosome arms 1p and 19q have been widely recognized as clinically relevant markers of glioma [16], and patients bearing IDH1 mutation are expected to have a better prognosis [17]. We then explored the correlation between *STOX1* expression and IDH1 mutation and found that IDH1-mutant patients exhibited a significantly higher expression of *STOX1* than IDH1 wild-type ones in both CGGA and TCGA datasets (Figure 1j and m). Moreover, *STOX1* expression was also found to be significantly higher in all IDH1-mutant gliomas in TCGA and GBMs in CGGA compared with their IDH1 wild-type counterparts (Figure 1l, n, and o). However, the difference was not significant in LGG patients of CGGA dataset (Figure 1k). The limited case number (*n* = 48) might be a possible reason. Considering that IDH1 wild-type GBMs are developed from IDH1 wild-type grade II or III astrocytomas or as *de novo*, which are originally different from IDH1-mutant astrocytomas and oligodendrogliomas or 1p19q codeleted oligodendrogliomas [18]. We compared *STOX1* expression of IDH1 wild-type GBMs with IDH1 wild-type astrocytomas and IDH1-mutant GBMs with IDH1-mutant astrocytomas. The results revealed that *STOX1* expression in IDH1 wild-type GBMs was significantly lower than that of IDH1 wild-type astrocytomas in both datasets (Figure A1a and b). Furthermore, in IDH1-mutant GBMs, *STOX1* was significantly downregulated compared to IDH1-mutant astrocytomas (Figure A1c and d). Altogether, these data suggested that the expression of *STOX1* was significantly downregulated in high-grade gliomas and that low expression of *STOX1* correlated with the glioma malignancy.

**Table 1:** Correlations between *STOX1* expression and clinicopathological characteristics in glioma patients in the CGGA

	*STOX1* expression		
Variables	Low (*n* = 162)	High (*n* = 163)	*P* value
Gender			0.1366
Male	108	95	
Female	54	68	
1p19q codeletion			0.7254
Yes	30	36	
No	128	123	
NA	4	4	
IDH1 status			<0.0001***
Wildtype	101	48	
Mutant	61	114	
NA	0	1	
Age			<0.0001***
<45	76	117	
≥45	86	46	
MGMT status			0.2957
Methylated	75	82	
Unmethylated	80	69	
NA	7	12	
Chemotherapy			0.5827
Yes	98	94	
No	52	60	
NA	12	9	
Radiotherapy			0.9964
Yes	122	123	
No	32	33	
NA	8	7	
WHO grade			<0.0001***
II	31	72	
III	30	49	
IV	98	41	
NA	3	1	
Histology			<0.0001***
A	12	44	
O	22	30	
AA	22	40	
AO	5	7	
GBM	98	41	
NA	3	1	

Data analyzed using chi-square test. ***P < 0.0001.

IDH1: isocitrate dehydrogenase 1; WHO: World Health Organization; *STOX1*: storkhead box 1; A: astrocytoma; O: oligodendroglioma; AA: anaplastic astrocytoma; AO: anaplastic oligodendroglioma; GBM: glioblastoma multiforme.

**Table 2:** Correlation between *STOX1* expression and clinicopathological characteristics in glioma patients in TCGA

	*STOX1* expression		
Variables	Low (*n* = 304)	High (*n* = 305)	*P* value
Gender			0.5114
Male	181	173	
Female	123	132	
1p19q codeletion			<0.0001***
Yes	100	51	
No	200	253	
NA	4	1	
IDH1 status			<0.0001***
Wildtype	177	48	
Mutant	125	253	
NA	2	4	
Age			<0.0001***
<45	87	197	
≥45	217	108	
WHO grade			<0.0001***
II	58	158	
III	120	121	
IV	126	26	
Histology			<0.0001***
A	6	49	
O	50	67	
AA	51	63	
AO	58	29	
OA	2	42	
AOA	11	29	
GBM	126	26	

Data analyzed using chi-square test. ***P < 0.0001.

IDH1: isocitrate dehydrogenase 1; WHO: World Health Organization; *STOX1*: storkhead box 1; A: astrocytoma; O: oligodendroglioma; AA: anaplastic astrocytoma; AO: anaplastic oligodendroglioma; OA: oligoastrocytoma; AOA: anaplastic oligoastrocytoma; GBM: glioblastoma multiforme.

### 3.2 Correlations between *STOX1* expression and prognosis in glioma

Next we evaluated the association between *STOX1* expression and clinicopathologic features of glioma patients in CGGA and TCGA using the Pearson’s chi-square test. In both datasets, patients were divided into *STOX1*-low and high subgroups using the median value of *STOX1*. Decreased *STOX1* expression was significantly associated with advanced WHO grades (Grade IV) (*P* < 0.0001), more aggressive pathological subtypes (GBM) (*P* < 0.0001), older age (≥45 years) (*P* < 0.0001), and less IDH1 mutation (*P* < 0.0001) (Tables 1 and 2). Univariate Cox regression analysis showed that decreased *STOX1* expression was significantly associated with worse overall survival in both datasets (Tables 3 and 4) (hazard ratio [HR]: 0.529, 95% confidence interval [CI]: 0.406–0.689, and *P* < 0.001 in CGGA and HR: 0.340, 95% CI: 0.247–0.466, and *P* < 0.001 in TCGA). Further multivariate Cox regression analyses revealed that *STOX1* downregulation predicted shorter overall survival of glioma patients (Tables 3 and 4) (HR: 0.731, 95% CI: 0.542–0.987, and *P* = 0.041 in CGGA and HR: 0.719, 95% CI: 0.469–1.104, and *P* = 0.132 in TCGA). These results indicated that low *STOX1* expression might serve as an independent indicator of poor prognosis in glioma patients.

**Table 3:** Univariate and multivariate Cox regression analyses for overall survival in glioma samples of the CGGA

	Univariate			Multivariate		
Variables	HR	95% CI	*P* value	HR	95% CI	*P* value
Gender	1.072	0.821–1.400	0.607	—	—	—
1p19q codeletion	0.196	0.124–0.309	<0.001***	0.301	0.167–0.544	<0.001***
IDH1 status	0.360	0.275–0.473	<0.001***	1.034	0.730–1.464	<0.001***
Age	0.532	0.408–0.693	<0.001***	0.871	0.644–1.177	0.369
MGMT status	0.832	0.637–1.086	0.176	—	—	—
Histology	1.675	1.518–1.847	<0.001***	—	—	—
Chemotherapy	1.321	1.000–1.743	0.050	0.743	0.544–1.016	0.063
Radiotherapy	0.671	0.495–0.909	0.010*	0.777	0.561–1.075	0.128
WHO grade	2.847	2.374–3.413	<0.001***	1.990	0.813–4.875	<0.001***
*STOX1* expression	0.529	0.406–0.689	<0.001***	0.724	0.535–0.981	0.037*

*P < 0.05 and ***P < 0.001.

IDH1: isocitrate dehydrogenase 1; MGMT: *O*[6]-methylguanine-DNA methyltransferase; WHO: World Health Organization; *STOX1*: storkhead box 1; HR: hazard ratio; CI: confidence interval.

**Table 4:** Univariate and multivariate Cox regression analyses for overall survival in glioma samples of TCGA

	Univariate			Multivariate		
Variables	HR	95% CI	*P* value	HR	95% CI	*P* value
Gender	0.999	0.742–1.346	0.997	—	—	—
1p19q codeletion	0.220	0.130–0.375	<0.001***	0.427	0.211–0.863	0.018*
IDH1 status	0.091	0.064–0.129	<0.001***	0.356	0.198–0.638	0.001**
Age	5.137	3.567–7.397	<0.001***	2.496	1.568–3.973	<0.001***
Histology	1.663	1.521–1.820	<0.001***	1.051	0.890–1.240	0.558
WHO grade	4.901	3.822–6.285	<0.001***	1.855	1.116–3.085	0.017*
*STOX1* expression	0.340	0.247–0.466	<0.001***	0.719	0.469–1.104	0.132

*P < 0.05; **P < 0.01; and ***P < 0.001.

IDH1: isocitrate dehydrogenase 1; MGMT: *O*[6]-methylguanine-DNA methyltransferase; WHO: World Health Organization; *STOX1*: storkhead box 1; HR: hazard ratio; CI: confidence interval.

**Figure 2:**
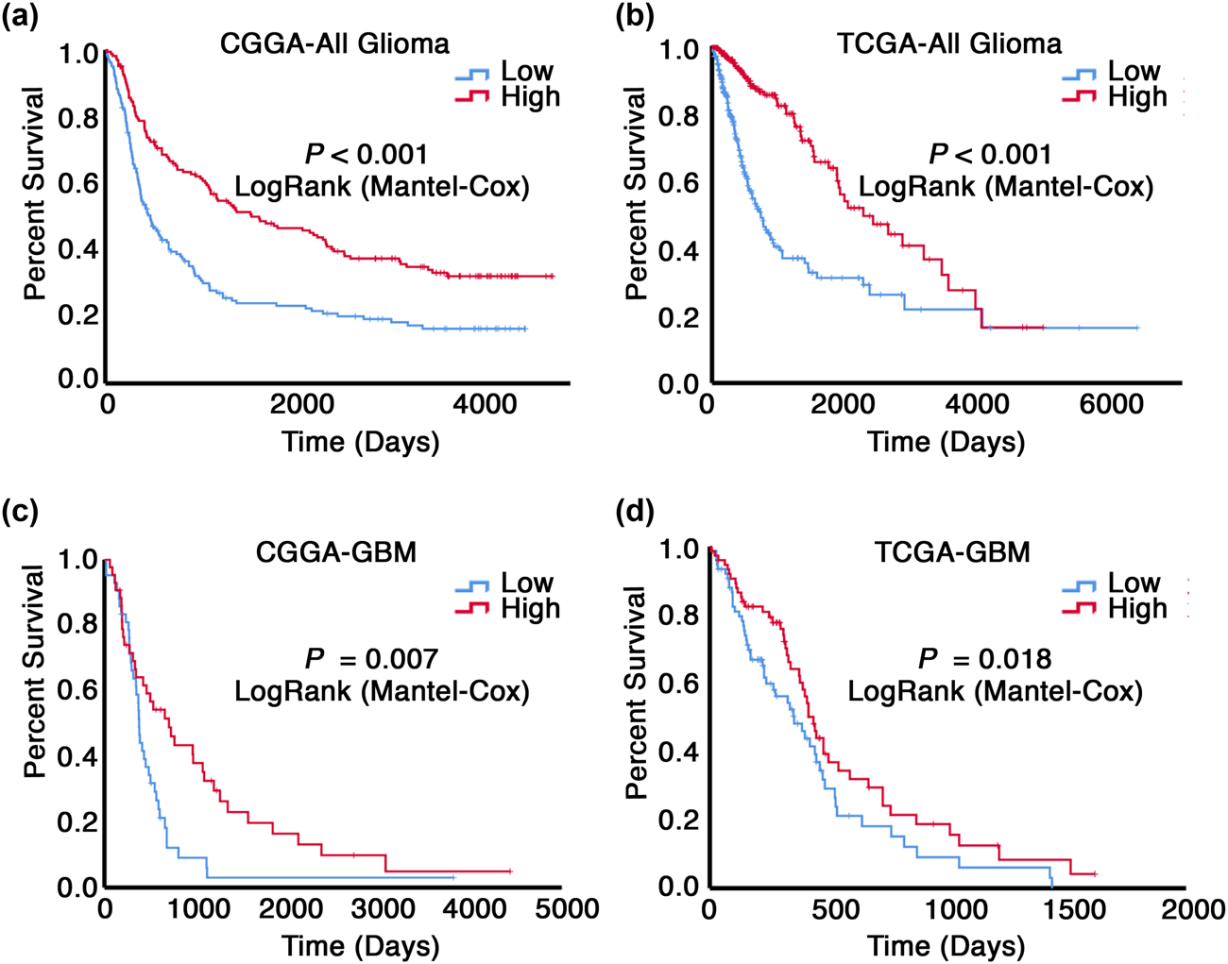
Kaplan–Meier analysis of overall survival based on *STOX1* expression levels in glioma patients. (a–d) Glioma patients with high *STOX1* expression had longer OS than those with low *STOX1* expression in all glioma cohort or GBM cohort alone in both CGGA and TCGA datasets. CGGA: Chinese Glioma Genome Atlas; TCGA: The Cancer Genome Atlas; OS: overall survival; GBM: glioblastoma multiforme.

**Figure A2:**
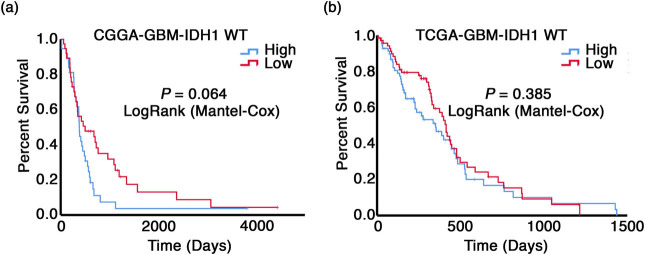
Kaplan–Meier analysis of overall survival based on *STOX1* expression in IDH1-WT GBMs. The difference in overall survival of the *STOX1*-low or -high IDH1-WT GBM patients was not statistically significant in both CGGA (a) and TCGA (b) datasets. GBM: glioblastoma multiforme; WT: wild type; CGGA: Chinese Glioma Cancer Atlas; TCGA: The Cancer Genome Atlas.

To further explore the correlation between *STOX1* expression and prognosis of glioma patients, Kaplan–Meier survival curve analysis together with a log-rank comparison was then employed to analyze the differences in the overall survival (OS) of *STOX1*-low and -high glioma patients in each dataset. As shown in Figure 2a–d, patients with high expression of *STOX1* had significantly longer OS than those with low *STOX1* expression in all gliomas or GBM cohort alone in both CGGA (All gliomas, *P* < 0.001 and GBM, *P* = 0.007) and TCGA (All gliomas, *P* < 0.001 and GBM, *P* = 0.018) datasets. Moreover, the relationship between *STOX1* expression and OS in IDH1 wild-type GBMs was also analyzed in CGGA and TCGA datasets. However, no significant differences were observed in both datasets (Figure A2a and b). To sum up, the data above suggest that downregulation of *STOX1* is associated with worse overall survival and may serve as a tumor biomarker in malignant glioma.

### 3.3 GSEA of *STOX1*-mediated signaling pathways in glioma

**Figure 3:**
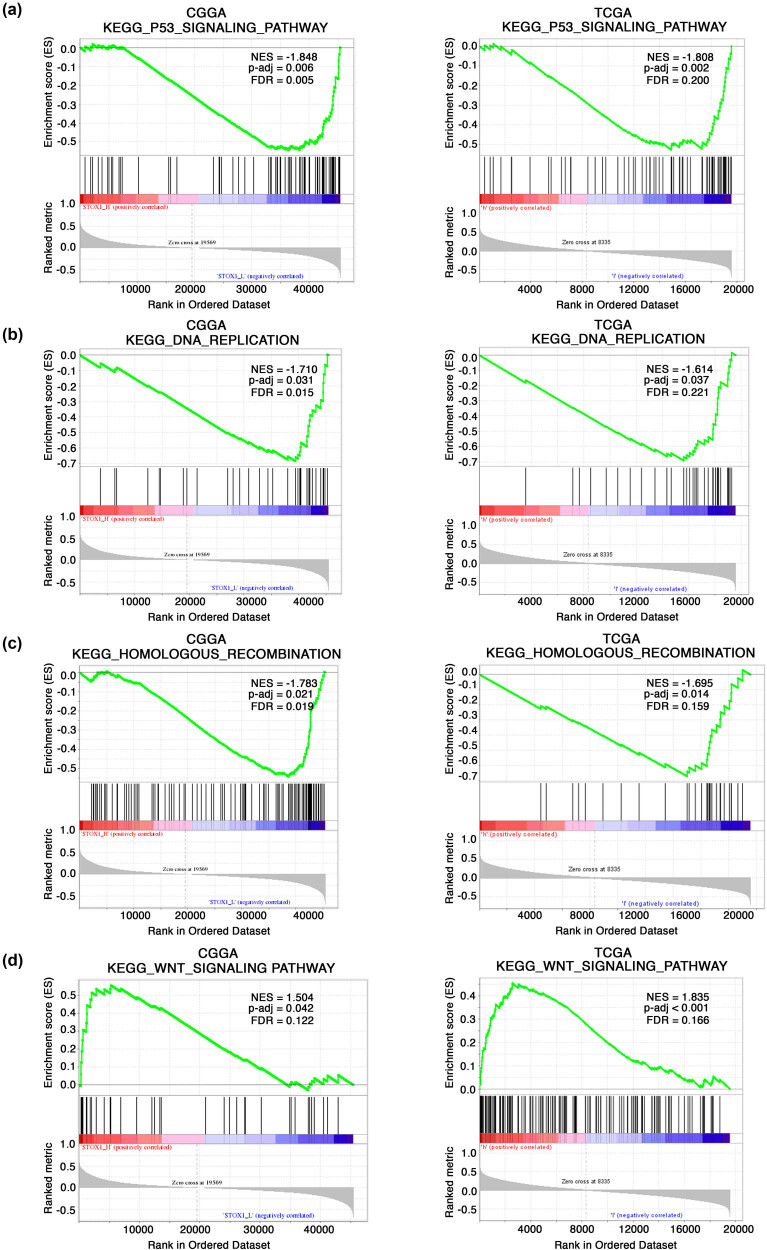
Enriched plots from gene set enrichment analysis (GSEA). Several pathways were differentially enriched in *STOX1*-related glioma including the p53 signaling pathway (a), DNA replication (b), homologous recombination (c), and Wnt signaling pathway (d) in both CGGA and TCGA datasets. CGGA: Chinese Glioma Genome Atlas; TCGA: The Cancer Genome Atlas; KEGG: Kyoto Encyclopedia of Genes and Genomes; NES: normalized enrichment score; FDR: false discovery rate.

To explore the potential functions of *STOX1* in glioma, GSEA was performed using primary data from CGGA and TCGA datasets. CGGA was used as a training set, while TCGA was included as a validation set. Significant differences (false discovery rate [FDR] < 0.25 and p.adjust [*P*] < 0.05) were demonstrated in the rich set of MsigDB (C2.all.v7.0.symbols.GMT) collection. Four gene sets were found to be significantly enriched and associated with the following pathways: P53_SIGNALING_PATHWAY (CGGA, *P* = 0.006, FDR = 0.005, and normalized enrichment score [NES] = −1.848 and TCGA, *P* = 0.002, FDR = 0.200, and NES = −1.808; Figure 3a); DNA_REPLICATION (CGGA, *P* = 0.031, FDR = 0.015, and NES = −1.710 and TCGA, *P* = 0.037, FDR = 0.221, NES = −1.614; Figure 3b); HOMOLOGOUS_RECOMBINATION (CGGA, *P* = 0.021, FDR = 0.019, and NES = −1.783 and TCGA, *P* = 0.014, FDR = 0.159, and NES = −1.695; Figure 3c), WNT_SIGNALING_PATHWAY (CGGA, *P* = 0.042, FDR = 0.122, and NES = 1.504 and TCGA, *P* < 0.001, FDR = 0.166, and NES = 1.835; Figure 3d). These results indicated that *STOX1* may regulate the P53 pathway, DNA replication, homologous recombination, and Wnt signaling pathway in glioma.

## 4 Discussion

In the present study, the association between *STOX1* expression and prognosis of glioma patients were evaluated based on the CGGA, TCGA, and Rembrandt datasets. We showed that *STOX1* expression was downregulated in high-grade (Grades III and IV) gliomas but not in Grade II tumors when compared to nontumor brain tissues and exhibited a grade-dependent decrease in all gliomas. Low *STOX1* expression could also independently predict a worse prognosis in glioma patients. To our knowledge, this is the first time to report *STOX1* expression and its prognostic value in cancer, and we infer that *STOX1* may function as a tumor suppressor in glioma. This hypothesis gains support from two recent studies in which retroviral gene-trap and functional characterization of primary mouse astrocyte clones under p53^–/–^ background found that *STOX1A* was one of the top two altered proteins [15] and *STOX1A* was a transcriptional suppressor of Math1 during cerebellar granule neurogenesis and medulloblastoma formation [14].

The *STOX1* gene is located on 10q22.1 and encodes a winged helix transcription factor which functionally and structurally resembles the Forkhead protein family [6,7]. Chromosome 10 harbors a number of important tumor suppressor genes including MMAC/PTEN (10q23) and DMBT1 (10q25), and loss of heterogeneity in this region could lead to tumorigenesis of primary brain tumors, especially primary GBMs [19,20]. Interestingly, the *STOX1* gene loci is close to these tumor suppressors on chromosome 10, and one may infer that grade-dependent downregulation of *STOX1* in glioma may result from loss of heterogeneity. However, this possibility could probably be ruled out because genetic alteration analysis demonstrated a <0.5% deletion of *STOX1* gene in 1,122 glioma patients (LGG, *n* = 516 and GBM *n* = 606) patients (cBioPortal, https://www.cbioportal.org) (data not shown), suggesting that loss of *STOX1* in glioma may be due to some upstream regulations instead. However, another study reported that *STOX1A* played a tumor-promoting role in human neuroblastoma cell SH-SY5Y and favored mitotic entry by binding directly to Cyclin B1 promoter [8], suggesting that *STOX1* may promote or suppress malignancy depending on tumor cell types. And since *STOX1B* may compete with *STOX1A* for the DNA-binding sites [5,7], the involvement of *STOX1B* or other *STOX1* isoforms may also contribute to these discrepancies, which needs further investigation.

Over the past decade, great advances have been achieved in understanding the molecular pathology of glioma and a series of molecular markers have been found helpful in the diagnosis, classification, and prognosis of glioma. IDH1 mutation is present in more than 70% of grade II and III gliomas and around 85% of secondary grade IV GBMs, which usually evolve from astrocytoma [1]. IDH mutation has also been found to be associated with a better prognosis and serve as an independent prognostic marker in glioma patients. Therefore, the status of IDH1 mutation has been incorporated into the WHO classification system in 2016 [21]. This is consistent with the univariate and multivariate Cox regression analyses in our study, which demonstrated that IDH1 mutation status was an independent favorable biomarker for overall survival of glioma patients in both CGGA and TCGA datasets. These findings are consistent with previous reports. We also found that low expression of *STOX1* was more often found in IDH1 wild-type gliomas, suggesting that *STOX1* may be functionally associated with IDH1 status.

1p/19q codeletion is detected in 80–90% of oligodendrogliomas, 50–70% of anaplastic oligodendrocytomas, 15% of diffuse astrocytomas, and only 5% of GBMs [22]. It is strongly associated with oligodendroglial histology and considered a diagnostic molecular biomarker for oligodendroglioma [22]. Clinical studies have also reported that patients with 1p/19q codeleted gliomas have a longer overall survival than those with non-codeleted tumors [23]. This is consistent with our results that 1p/19q codeletion could also serve as an independent predictive biomarker for poorer overall patient survival in both datasets. Moreover, we found that low expression of *STOX1* was more frequently present in 1p/19q codeleted gliomas in TCGA but not in CGGA. This discrepancy may result from the relatively small sample size in CGGA dataset (*n* = 325) compared to TCGA dataset (*n* = 609). There are also other molecular markers that have been studied extensively, such as EGFR, MGMT, and so on. The 2016 WHO classification system of brain tumors, for the first time incorporated these molecular markers for more precise diagnosis of glioma [24]. However, targeted therapies based on these molecular markers have turned out be unencouraging [25]. Identification of novel biomarkers is necessary and *STOX1* may be a promising candidate.

Functional analysis of *STOX1* in glioma with GSEA revealed that enriched gene sets were associated with p53 signaling pathway and DNA replication. p53 is a transcription factor responsible for multiple cellular processes including cell metabolism, cell cycle transition, DNA repair and replication, senescence, and apoptosis [26]. When activated, p53 transcriptionally promotes p21 expression, which in turn not only induces G1 phase arrest and inhibits DNA replication by inhibiting Cyclin D/CDK4 and Cyclin E/CDK2 but also inhibits mitosis at G2/M phase by inhibiting Cyclin B/cdc2 [26]. *STOX1* has also been found to be involved in cell cycle regulation. Oudejans et al. first discovered that *STOX1A* promoted mitotic entry through directly binding to Cyclin B1 promoter in human neuroblastoma cell SH-SY5Y [8]. Later, Liu and colleagues found that knockdown of *STOX1* induced depression of S phase in rat pulmonary arterial smooth muscle cells [9]. Accordingly, *STOX1* may also regulate cell cycle transition and DNA replication in glioma cells directly or with the involvement of p53, which needs further investigation. In addition, the enriched gene sets were also related to homologous recombination and Wnt signaling pathway. Homologous recombination is a DNA repair pathway and its deficiency could sensitize cancer cells to chemo/radiotherapies [27]. Specifically, homologous-recombination deficient cells exhibited better sensitivity to poly-ADP ribose polymerase (PARP) inhibitors [27]. Noticeably, chemotherapy such as alkylating agents and radiotherapy for glioma both target DNA damage [1], and manipulation of *STOX1* could potentially contribute towards the efficacies of PARP inhibitor treatment or chemo/radiotherapies alone or in combination in glioma. Wnt signaling pathway plays a pivotal role in the regulation of cell growth, cell development, and differentiation of stem cells; constitutive activation of this pathway has been found in many human cancers [28,29]. Notably, Wnt2B, an important member and activator of the Wnt signaling pathway, has been implicated in multiple cancers [30,31] and was found to be a direct target of *STOX1* in SK-N-SH cells [32]. Altogether, all these enriched gene sets are related to cancer and further investigation on the mechanistic relationships between *STOX1* and these pathways could provide novel insights into the pathogenesis and treatment of glioma.

This study has several limitations. First, due to the lack of glioma transcriptome datasets which distinguish the expression profiles of *STOX1* transcript isoforms, *STOX1* was considered as a single entity in the current study. Second, protein expression levels of *STOX1* in gliomas were not investigated due to insufficient glioma specimens in our hospital. This work should be required in the future through immunohistochemical staining or western blot on a large sample size of glioma specimens. Last but not least, our bioinformatic analysis had not been verified experimentally. However, one may argue that bioinformatic studies, which analyze multiple public datasets with large sample size of patient samples, could actually provide more comprehensive understanding of gene expression profiles, associations between gene expression and clinicopathological characteristics, and most importantly, potential functions that genes are most possibly and also unexpectedly involved in. For example, large-scale transcription-based analysis using integrated data from TCGA opened a new era for the WHO classification system for GBM in 2016 [33]. Similar great success has also been achieved in recent cancer studies published in big journals [34,35]. Nevertheless, additional molecular experiments would no doubt contribute to the understanding.

## 5 Conclusion

This study, through the analysis of several database data, for the first time provides comprehensive evidence that *STOX1* is downregulated in high-grade gliomas, correlates with glioma grade, and might serve as an independent prognostic biomarker for glioma patients. *STOX1* may be one of the negative modulators of glioma and downregulation of *STOX1* may lead to the malignant progression of glioma. Although the roles of *STOX1* in glioma have not yet been clarified, our study predicts that *STOX1* may be used as a biomarker to assist in the diagnosis and treatment of glioma, and it could also be used as a prognostic marker for glioma patients.
